# Label-Free Electrochemical Dopamine Biosensor Based on Electrospun Nanofibers of Polyaniline/Carbon Nanotube Composites

**DOI:** 10.3390/bios14070349

**Published:** 2024-07-18

**Authors:** Chanaporn Kaewda, Saengrawee Sriwichai

**Affiliations:** Department of Chemistry, Faculty of Science, Chiang Mai University, Chiang Mai 50200, Thailand; chanaporn_kaewda@cmu.ac.th

**Keywords:** conducting polymer, biosensor, electrochemical detection, dopamine, polyaniline, poly(3-aminobenzylamine), carbon nanotube, SDG3: good health and well-being

## Abstract

The development of conducting polymer incorporated with carbon materials-based electrochemical biosensors has been intensively studied due to their excellent electrical, optical, thermal, physical and chemical properties. In this work, a label-free electrochemical dopamine (DA) biosensor based on polyaniline (PANI) and its aminated derivative, i.e., poly(3-aminobenzylamine) (PABA), composited with functionalized multi-walled carbon nanotubes (f-CNTs), was developed to utilize a conducting polymer as a transducing material. The electrospun nanofibers of the composites were fabricated on the surface of fluorine-doped tin oxide (FTO)-coated glass substrate under the optimized condition. The PANI/f-CNTs and PABA/f-CNTs electrospun nanofibers were characterized by attenuated total reflectance–Fourier transform infrared (ATR-FTIR) spectroscopy, X-ray photoelectron spectroscopy (XPS), scanning electron microscopy (SEM) and transmission electron microscopy (TEM), which confirmed the existence of f-CNTs in the composites. The electroactivity of the electrospun nanofibers was investigated in phosphate buffer saline solution using cyclic voltammetry (CV) before being employed for label-free electrochemical detection of DA using differential pulse voltammetry (DPV). The sensing performances including sensitivity, selectivity, stability, repeatability and reproducibility of the fabricated electrospun nanofiber films were also electrochemically evaluated. The electrochemical DA biosensor based on PANI/f-CNTs and PABA/f-CNTs electrospun nanofibers exhibited a sensitivity of 6.88 µA·cm^−2^·µM^−1^ and 7.27 µA·cm^−2^·µM^−1^ in the linear range of 50–500 nM (R^2^ = 0.98) with a limit of detection (LOD) of 0.0974 µM and 0.1554 µM, respectively. The obtained DA biosensor showed great stability, repeatability and reproducibility with precious selectivity under the common interferences, i.e., glucose, ascorbic acid and uric acid. Moreover, the developed electrochemical DA biosensor also showed the good reliability under detection of DA in artificial urine.

## 1. Introduction

Low levels of dopamine (DA) or 3,4-dihydroxyphenylethylamine, which is one of the important neurotransmitters in catecholamine family and naturally exists in human central nervous system, can cause various neurological diseases such as Parkinson’s disease, fatigue in multiple sclerosis, depression, psychiatric and other neurological disorders [1,2,3,4]. In addition, the decreasing of DA can result in the abandonment of effortful reward-seeking behavior and retardment of cognitive flexibility [2,5]. In fact, the range of DA concentrations in biological systems or organisms can be from 10^−7^ to 10^−3^ M [6]. Hence, the development of biosensors concerning the correct determinations of DA amount has been extensively focused. There are many recent studies that reported the development of high performance DA biosensors, including the silicon nanowire field-effect transistor biosensors for detection of DA released from living PC12 cells [7], the 3D-printed carbon microelectrodes with controllable geometries and sizes for detection of DA in a brain slice and in vivo [8], the dual-nanopore biosensor based on DNA aptamer recognition for detection of intracellular DA and DA efflux (extracellular dopamine) in a single pheochromocytoma (PC12) cell [9] and the organic electrochemical transistor based on poly(3,4-ethylenedioxythiophene)-poly(styrenesulfonate) or PEDOT:PSS for detection of DA in living rat brain [10]. Electrochemical methods such as cyclic voltammetry (CV), amperometry or differential pulse voltammetry (DPV) provide many advantages over other common analytical techniques such as fluorescence spectroscopy, high-performance liquid chromatography or gas chromatography due to their simple operation process, low cost and rapid analysis [11,12,13]. Moreover, DA is an electroactive molecule which can be easily detected by electrochemical methods [6]. However, the electrochemical detection of DA in biological systems can be interfered by the presence of high concentrations of ascorbic acid (AA), uric acid (UA) or glucose (Glu) [14,15,16]. To overcome this problem, the development of electrochemical DA biosensors with high sensitivity and selectivity has been strongly considered. One of the most effective approaches is to modify the conventional working electrode with the electroactive and selective materials, such as conducting polymers, nanomaterials or their composites, to improve the sensitivity and selectivity for DA measurement under common interferences.

Conducting polymers such as polyaniline, polythiophene and polypyrrole are the most promising electroactive materials for electrochemical biosensor applications [17,18,19,20,21,22]. The copolymer of 3,4-ethylenedioxythiophene (EDOT) and 2H-thieno [3,4-b][1,4]dioxepin-3,3(4H)-diacetic acid (ProDOT–(COOH)_2_) was first used for the detection of adenosine and mucin-1 with good sensitivity, selectivity and stability [17,22]. The molecularly imprinted polymers electrochemical biosensor based on poly(3-aminophenylboronic acid) (PAPBA) was also developed for specific detection of epinephrine in real human serum [19]. In addition, the flexible DA biosensor with high sensitivity and selectivity was fabricated from graphene oxide/PEDOT:PSS (GO/PEDOT:PSS) composite [20]. Recently, polyaniline and its derivatives incorporated with nanomaterials such as carbon quantum dots (CQDs) [13], graphene oxide (GO) [18], gold (Au) [23], carbon nanomaterials [16,24,25] and iron oxide (Fe_2_O_3_) [26] are promising materials for electrochemical biosensors. Carbon nanotubes (CNTs) are one class of carbon nanomaterials that have been widely used for incorporation with various conducting polymers due to how they can promote electron transfer and increase electrode surface area [16,27]. In addition, the functionalization of CNTs can serve as the preferable method to improve the dispersion and compatibility of CNTs with conducting polymers [24]. Hence, we used functionalized multi-walled carbon nanotubes (f-CNTs) for the improvement of the conducting polymer properties in this study.

The nanofibers with diameters ranging from about ten to one hundred nanometers have been considered as the alternative choice for adjusting surface morphology and improving the electrode surface area which can affect the biosensor performances. Electrospinning is the versatile method that could produce the continuous ultrathin nanofibers with simple and efficient production [28,29,30,31]. This method has been widely used for the fabrication of the conducting polymers-based nanofibers in various applications including drug release, tissue engineering and biosensors [32,33,34,35]. The obtained electrospun nanofibers with a high surface area to volume ratio and porous structure can serve as higher sensitivity and selectivity biosensors than flat surface biosensors [36,37]. Therefore, in this study, electrospinning was employed for the fabrication of the label-free electrochemical dopamine biosensor based on conducting polymer composites.

**Scheme 1:**
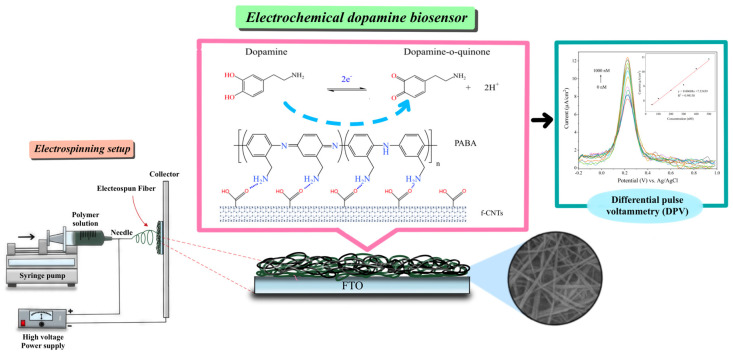
Schematic illustration of the fabrication of the label-free electrochemical dopamine biosensor based on the electrospun nanofibers of PANI/f-CNTs and PABA f-CNTs composites.

Recently, we fabricated the PABA film using a layer-by-layer deposition technique for detection of DA [38]. However, the performance of the obtained DA biosensor was still unsatisfactory. Therefore, this study aims to develop a label-free electrochemical dopamine biosensor based on the electrospun nanofibers of polyaniline (PANI) and its aminated derivative, i.e., poly(3-aminobenzylamine) (PABA), composited with f-CNTs for the detection of low linear ranges with high sensitivity. First, the PANI/f-CNTs and PABA/f-CNTs composites were synthesized by in situ chemical oxidation polymerization using ammonium persulfate as an oxidizing agent. The obtained composites were then employed for fabrication of the electrochemical dopamine biosensor based on PANI/f-CNTs and PABA/f-CNTs electrospun nanofibers on fluorine-doped tin oxide (FTO)-coated glass substrate under optimized conditions. The electrospun nanofibers were characterized by attenuated total reflectance–Fourier transform infrared (ATR-FTIR) spectroscopy, X-ray photoelectron spectroscopy (XPS), scanning electron microscopy (SEM) and transmission electron microscopy (TEM). To study the performances of the label-free electrochemical dopamine biosensor, the fabricated PANI/f-CNTs and PABA/f-CNTs electrospun nanofibers on FTO-coated glass substrate were used as working electrodes in the electrochemical measurement of DA using DPV. The presence of f-CNTs in the composites exhibited the improvement of sensitivity, selectivity, reproducibility, repeatability and stability of the DA biosensor. Moreover, the developed label-free electrochemical dopamine biosensor was successfully applied for the detection of DA in artificial urine, which may be applied in human health diagnoses in the future. Scheme 1 presents the building-up of the label-free electrochemical dopamine biosensor based on the electrospun nanofibers of PANI/f-CNTs and PABA f-CNTs composites.

## 2. Experimental Section

### 2.1. Reagents and Materials 

Dopamine (DA), aniline (ANI), 3-Aminobenzyl amine (ABA), glucose (Glu), dimethyl formamide (DMF), polyacrylonitrile (PAN), potassium chloride (KCl), phosphate buffer saline (PBS) tablets and fluorine-doped tin oxide (FTO)-coated glass substrate were purchased from Sigma-Aldrich (Darmstadt, Germany). Ascorbic acid (AA) was purchased from Poch (Gliwice, Poland). Uric acid (UA) was purchased from Bio Basic (Markham, ON, Canada). Potassium hexacyanoferrate (K_3_Fe(CN)_6_) was purchased from Scharlau (Barcelona, Spain). Ammonium persulfate was purchased form RCI Labscan (Bangkok, Thailand). The artificial urine was purchased from Pickering Laboratories (Mountain View, CA, USA). All chemicals were used without further purification. All aqueous solutions were prepared with DI water. The FTO-coated glass substrate was cleaned prior to use. The PANI and PABA were synthesized by chemical oxidation polymerization using ammonium persulfate as an oxidant [38,39]. The f-CNTs was prepared by functionalization of multi-walled carbon nanotubes in nitric acid and sulfuric acid (1:3) [24].

### 2.2. Instrumentation 

The chemical constituents of the fabricated electrospun nanofibers were investigated using ATR-FTIR spectroscopy (Bruker Tensor 27, Billerica, MA, USA) and X-ray photoelectron spectroscopy (XPS; AXIS ultra DLD spectrometer, Manchester, UK). The surface morphology was studied using scanning electron microscopy (SEM; JEOL JSM–6335F, Tokyo, Japan) and transmission electron microscopy (TEM; JEOL JEM-2010, Tokyo, Japan). All electrochemical experiments including CV and DPV techniques were performed using eDAQ; the ED410 e-corder 410 potentiostat in the 3-electrode system consisted of the FTO-coated glass substrate as working electrode, platinum wire as counter electrode and Ag/AgCl (in 3 M KCl) as reference electrode. The electrochemical characteristics of the fabricated electrospun nanofibers of PANI/f-CNTs and PABA/f-CNTs composites were performed in a PBS solution containing 0.1 M KCl and 0.5 mM K_3_Fe(CN)_6_ as a redox mediator at various scan rates (10–100 mV/s) in the potential range of −0.2–1.0 V using CV technique prior to use as the label-free electrochemical dopamine biosensor.

### 2.3. Fabrication of PANI/f-CNTs and PABA/f-CNTs Electrospun Nanofiber Films

The PANI/f-CNTs and PABA/f-CNTs composites were firstly synthesized by in situ chemical oxidation polymerization of 50 mM monomers (anline and ABA, respectively) and 0.6% *w*/*v* f-CNTs in an aqueous solution using ammonium persulfate as an oxidizing agent [40]. The electrospun nanofiber films of the PANI/f-CNTs and PABA/f-CNTs composites were then fabricated on the FTO-coated glass substrate through an electrospinning technique. For the electrospinning experiment, the chemically synthesized composite powders (1% *w*/*v*) and PAN (3% *w*/*v*) were dissolved in DMF. The mixtures were sonicated for 1 h and centrifuged at 4000 rpm for 1 h prior to use for fabrication of the PANI/f-CNTs and PABA/f-CNTs composite nanofibers. The optimized electrospinning condition was a voltage of 15 kV, a feeding rate of 1.0 mL/h, a distance between the syringe and the collector (i.e., FTO-coated glass substrate) of 10 cm and a period of time of 10 min. The fabricated electrospun nanofiber films were further employed for label-free electrochemical detection of DA.

### 2.4. Electrochemical Detection of Dopamine 

The obtained electrospun nanofibers of PANI/f-CNTs and PABA/f-CNTs composites on FTO-coated glass substrate were used as a working electrode for electrochemical detection of DA in the PBS solution containing 0.5 mM K_3_Fe(CN)_6_ and 0.1 M KCl at a scan rate of 5 mV/s in the potential range of −0.2–1.0 V using DPV technique. The sensitivity performance of the electrospun nanofiber films was studied by successive adding of various concentrations of DA (50–2000 nM for PANI and PABA, 50–1000 nM for PANI/f-CNTs and PABA/f-CNTs). For the selectivity experiment, the DPV responses upon adding 1 mM of the common interferences, i.e., glucose, ascorbic acid and uric acid, were investigated. The reproducibility was also evaluated by using three similar independently electrospun nanofiber films via the addition of 1 mM DA into the buffer solution. Furthermore, the repeatability was studied via successively adding 1 mM DA for 10 cycles of DPV scanning. The stability of the electrospun nanofiber films was performed for up to 30 days. 

### 2.5. Application of the Obtained Label-Free DA Biosensor in Artificial Urine 

For the detection of DA in artificial urine, the electrochemical detection of DA at concentrations of 0.15, 0.25 and 0.35 µM was performed in 5% *v*/*v* of artificial urine in PBS solution containing 0.5 mM K_3_Fe(CN)_6_ and 0.1 M KCl using the electrospun nanofibers of PANI/f-CNTs and PABA/f-CNTs composites on FTO-coated glass substrate as the working electrode. The DPV responses for detection of DA in artificial urine were recorded in the potential range of −0.2–1.0 V at a scan rate of 5 mV/s using three similar independent electrodes. The response DPV peak currents for each concentration in the artificial urine were employed for calculation of percentage recovery.

## 3. Results and Discussion

### 3.1. Characterization of Electrospun Nanofiber Films 

**Figure 1:**
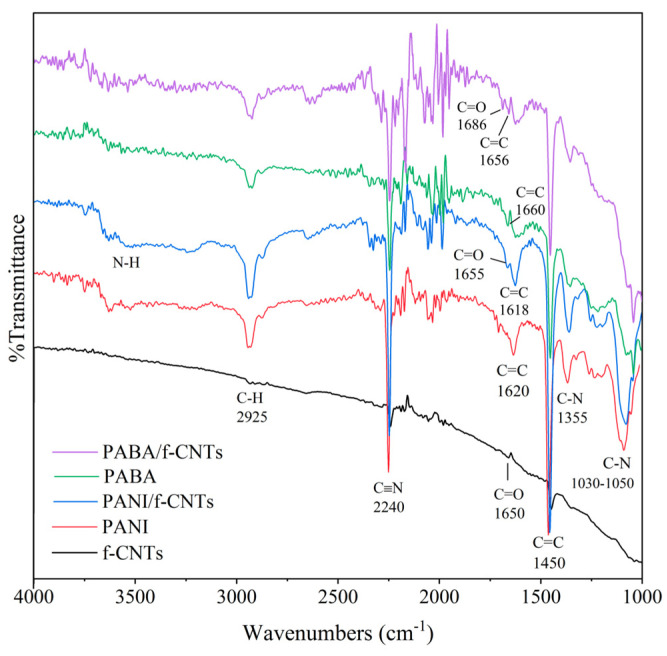
ATR-FTIR spectra of f-CNTs, PANI, PANI/f-CNTs, PABA and PABA/f-CNTs electrospun nanofiber films.

The fabricated PANI/f-CNTs and PABA/f-CNTs electrospun nanofiber films on FTO-coated glass substrate were characterized by ATR-FTIR spectroscopy, XPS, SEM and TEM. The ATR-FTIR spectroscopy utilized for investigation the functional groups and chemical constituents of the electrospun nanofiber films on FTO-coated glass substrate. As shown in Figure 1, the aromatic C-H stretching vibrations of f-CNTs, PANI, PANI/f-CNTs, PABA and PABA/f-CNTs exhibited peaks at about 2925 cm^−1^. The peaks at 1620 and 1450 cm^−1^ were assigned to the C=C stretching of quinoid and benzenoid rings, respectively, for PANI, whereas the PABA showed the C=C stretching of quinoid and benzenoid rings at 1660 and 1450 cm^−1^, respectively. These peaks were shifted to lower wavenumbers for PANI/f-CNTs and PABA/f-CNTs, as seen in Figure 1, which could imply the incorporation of PANI and PABA with f-CNTs [41]. The C-N stretching vibrations of PANI and PABA structures in PANI, PANI/f-CNTs, PABA and PABA/f-CNTs fibers presented the peaks at 1355 and 1030–1050 cm^−1^ [16,40,42]. The broad brand at around 3500 cm^−1^ indicated the N-H stretching vibration in PANI and PABA structures [13,24,38]. The significant C-O stretching vibration of COOH groups on the surface of f-CNTs showed the peak at about 1650 cm^−1^ for f-CNTs, 1655 cm^−1^ for PANI/f-CNTs and 1686 cm^−1^ for PABA/f-CNTs [43,44]. Shifts of the C-O peak were observed in the composite, which could again confirm the interaction of f-CNTs and the polymers. The peak at 2240 cm^−1^ attributed to the C≡N group in PAN which was used as a blending agent for the electrospinning process [45]. The FTIR spectra of the PANI/f-CNTs and PABA/f-CNTs electrospun nanofiber films could confirm the appearance of f-CNTs in the composite films. Furthermore, other techniques, i.e., XPS, SEM and TEM were also used to confirm the presence of f-CNTs in the PANI/f-CNTs and PABA/f-CNTs electrospun nanofiber films.

**Figure 2:**
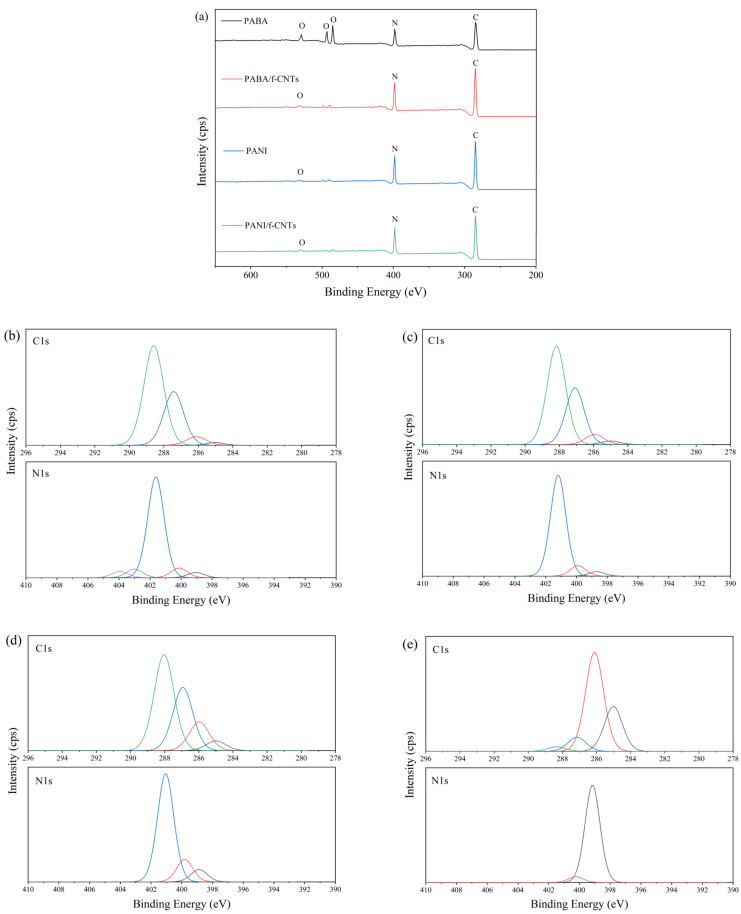
XPS (**a**) survey spectra and high-resolution spectra of (**b**) PANI, (**c**) PANI/f-CNTs, (**d**) PABA and (**e**) PABA/f-CNTs electrospun nanofiber films.

XPS also provides the chemical compositions of the electrospun nanofiber films as seen in Figure 2. The survey spectra of the PANI, PANI/f-CNTs, PABA and PABA/f-CNTs electrospun nanofiber films in Figure 2a presented the C1s and N1s peaks at about 285 and 398 eV, respectively. In case of the PABA film, the O1s peaks of surface oxygen at about 500 and 550 eV were clearly observed, which probably arose from the hydrolysis of PABA with water molecules upon the preparation process and storage contamination [41,46]. The high-resolution C1s and N1s spectra of PANI and PANI/f-CNTs films are shown in Figure 2b,c, respectively. The C1s peaks of PANI film presented at binding energy of 285.5, 286.1, 287.4 and 288.6 eV, which contributed to C=C, C-N, C-N^+^/C=N^+^ and C=N, respectively, in PANI structure. The PANI/f-CNTs composite film exhibited the C1s peaks which were identical to the PANI film. However, the N1s spectra of PANI and PANI/f-CNTs are different. The N1s spectrum of PANI film showed 5 peaks at 399.0, 400.1, 401.6, 403.0 and 403.9 eV, which originated from neutral imine (=N-), neutral amine (-NH-) or delocalized polaron-type structures (-NH^+^), -N^+^-, localized bipolaron type structures (=NH^+^-) and protonated imine (-NH_2_^+^), respectively [46,47,48]. The disappearance of =NH^+^- and -NH_2_^+^ components was observed in the PANI/f-CNTs film which indicated the interaction of PANI and f-CNTs in the fabricated composite film. In the case of the PABA (Figure 2d) and PABA/f-CNTs (Figure 2e) films, the C1s spectra of the PABA and PABA/f-CNTs films exhibited the identical C=C, C-N, C-N^+^/C=N^+^ and C=N components at binding energies of 285.0, 286.0, 286.8 and 288.0 eV, respectively. The ratio of each components in PABA/f-CNTs film was obviously changed which could indicate the interaction of PABA and f-CNTs in the composite film. The N1s spectrum of PABA at binding energies of 398.9, 399.8 and 401.1 eV contributed to –N=, -NH-/-NH_2_- and protonated primary amine (-NH_3_^+^) in the PABA structure [23,49,50]. The protonated primary amine (-NH_3_^+^) in PABA/f-CNTs film disappeared, which could confirm the interaction of PABA and f-CNTs in PABA/f-CNTs composite. Moreover, the ratio of –N=/-NH- peak obviously increased, indicating that the quinoid structure in PABA increased, which could imply that the doping level of PABA increased after being incorporated with f-CNTs [23,40]. The ratio of –N=/-NH- peaks were calculated to be 0.535 for PANI, 0.432 for PANI/f-CNTs, 0.552 for PABA and 16.871 for PABA/f-CNTs. According to XPS results, it is therefore concluded that the PANI/f-CNTs and PABA/f-CNTs composites could be successfully formed in this study.

**Figure 3:**
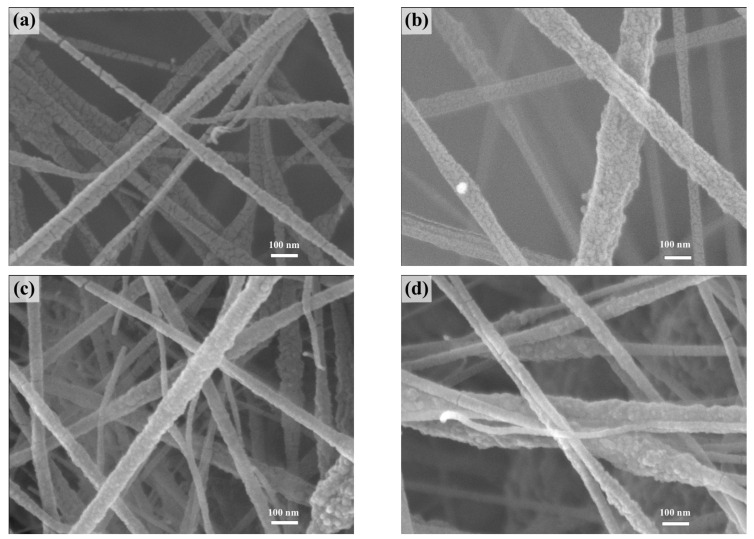
SEM images of (**a**) PANI, (**b**) PANI/f-CNTs, (**c**) PABA and (**d**) PABA/f-CNTs electrospun nanofiber films.

**Figure 4:**
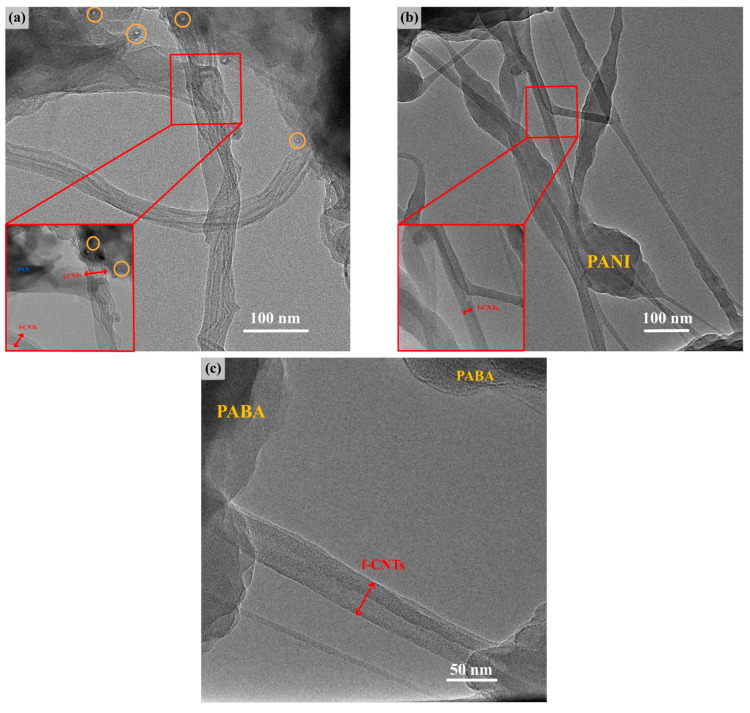
TEM images of (**a**) f-CNTs, (**b**) PANI/f-CNTs and (**c**) PABA/f-CNTs electrospun nanofiber films. Orange cycles referred to the carbon nanotube end; Large red rectangle is the expansion part of the small red rectangle for clearly seen the f-CNTs.

To study the morphology of the fabricated electrospun nanofiber films, SEM was performed in top view as presented in Figure 3. The surfaces of the electrospun nanofibers were observed to be uniform and bead-free. The average diameters of the PANI (Figure 3a), PANI/f-CNTs (Figure 3b), PABA (Figure 3c) and PABA/f-CNTs (Figure 3d) electrospun nanofibers were investigated to be 84.20 ± 16.39, 102.98 ± 24.85, 74.20 ± 19.79 and 77.94 ± 22.15 nm, respectively. The composite fiber sizes increased, which could confirm the incorporation of f-CNTs in PANI and PABA structures [13,24]. In addition, as shown in Figure 4, TEM images presented the appearance of f-CNTs (Figure 4a) in the electrospun composite nanofibers of PANI/f-CNTs (Figure 4b) and PABA/f-CNTs (Figure 4c). Hence, from both SEM and TEM results, it could be again confirmed the formation of PANI/f-CNTs and PABA/f-CNTs composites.

**Figure 5:**
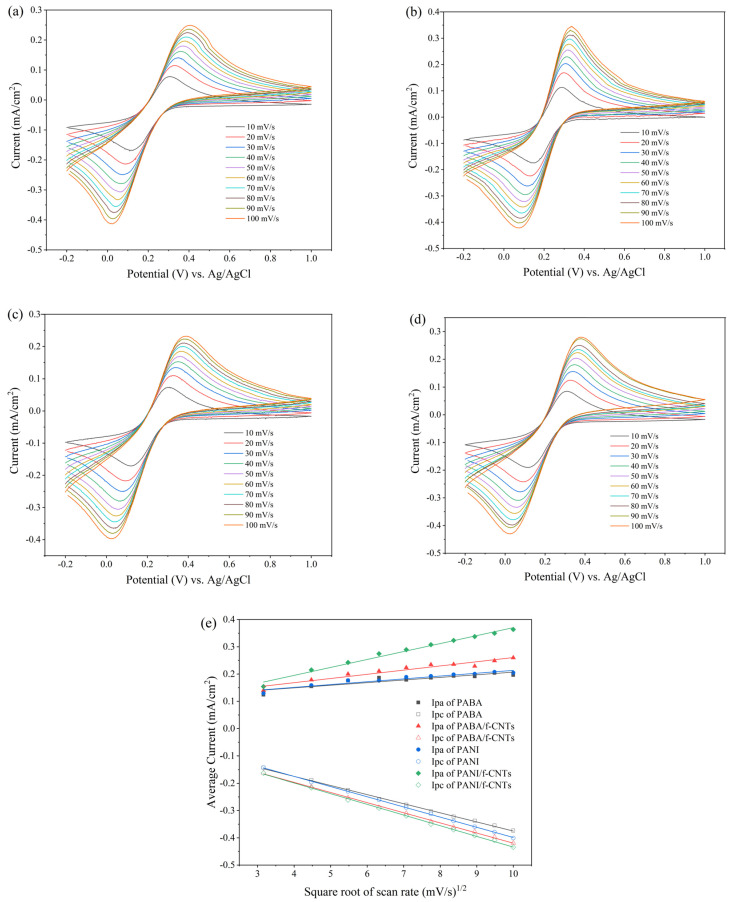
Cyclic voltammograms of (**a**) PANI, (**b**) PANI/f-CNTs, (**c**) PABA and (**d**) PABA/f-CNTs electrospun nanofiber films at different scan rates in PBS solution containing 0.5 mM K_3_Fe(CN)_6_ and 0.1 M KCl, and (**e**) linear plots between peak currents (I_p_) and square root scan rates.

In addition, prior to use as a label-free electrochemical DA biosensor, the electroactivity of the electrospun nanofiber films was studied in a PBS solution containing 0.5 mM K_3_Fe(CN)_6_ and 0.1 M KCl at various scan rates (10–100 mV/s) in the potential range of −0.2–1.0 V using CV technique. Figure 5 presents the CV curves (Figure 5a–d) with the corresponding linear relationship of the peak currents or I_c_ (anodic peak current, I_pa_ and cathodic peak current, I_pc_) and square root of the scan rates (v^1/2^) in Figure 5e. As can be seen from Figure 5a–d, the CV responses of the fabricated electrospun nanofiber films were broad, which indicated the quasireversible electron transfer process. The linear relationship of I_p_ and v^1/2^ (Figure 5e) indicated the diffusion control of the electrode surface [38,51]. The electrochemical surface area was then calculated from the Randles–Sevcik equation at the angular coefficient of the linear plot [13,52]. Table 1 shows the calculated electrochemical surface area of both cathodic (A_cathodic_) and anodic (A_anodic_) scans with the average difference between anodic and cathodic peak voltage (ΔE_a,c_) of the fabricated electrospun nanofiber films. The composite films exhibited a higher electrochemical surface area than the polymer films, which could imply that the addition of f-CNTs could enhance the electrochemical performance of the composite films. The ΔE_a,c_ that could be obtained from the CV responses were large, as shown in Table 1. In addition, the composite films presented lower values of ΔE_a,c_ than that of the polymer film. This could again imply that the addition of f-CNTs could enhance the electrochemical performance of the composite films. 

### 3.2. Electrochemical Detection of Dopamine

**Figure 6:**
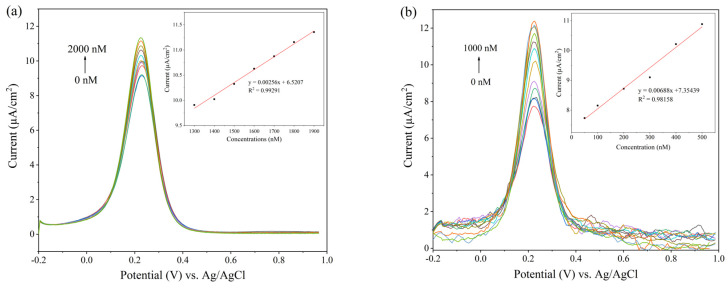

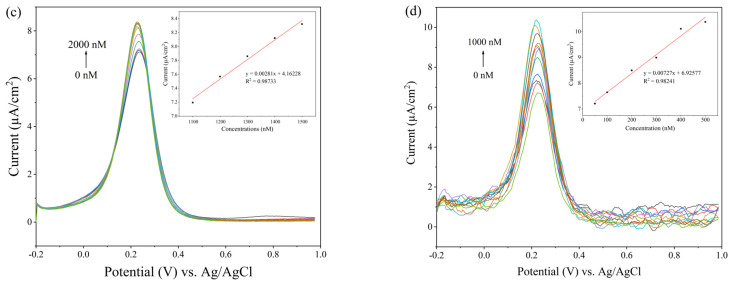
Differential pulse voltammograms of (**a**) PANI, (**b**) PANI/f-CNTs, (**c**) PABA and (**d**) PABA/f-CNTs electrospun nanofiber films upon successive addition with increasing concentration of dopamine in PBS solution containing 0.5 mM K_3_Fe(CN)_6_ and 0.1 M KCl ((insets) corresponding linear calibration curves of peak currents and DA concentrations).

**Scheme 2:**
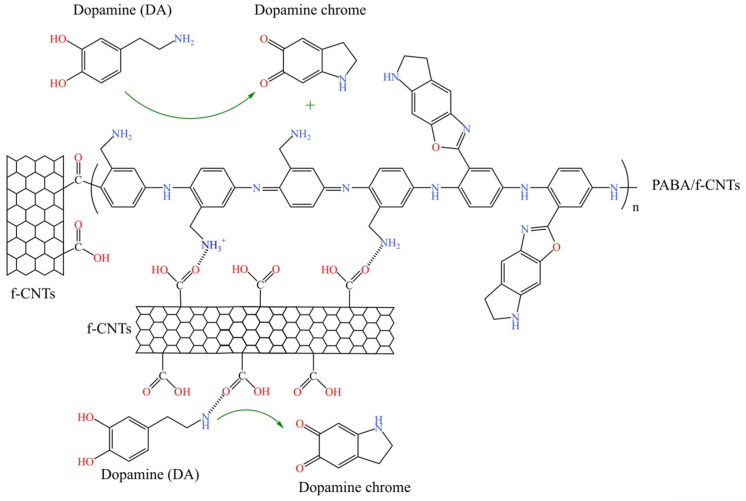
Proposed mechanism for detection of DA using PABA/f-CNTs electrospun film.

The obtained PANI/f-CNTs and PABA/f-CNTs electrospun nanofiber films were employed for the label-free electrochemical detection of dopamine at various concentrations (50–2000 nM for PANI and PABA, 50–1000 nM for PANI/f-CNTs and PABA/f-CNTs) using DPV technique at a potential range of −0.2 to 1.0 V and a scan rate of 5 mV/s in PBS solution containing 0.5 mM K_3_Fe(CN)_6_ and 0.1 M KCl. The DPV responses with corresponding calibration curves in the linear ranges for detection of DA are presented in Figure 6. The oxidation peak of the electrospun nanofiber films in PBS solution containing 0.5 mM K_3_Fe(CN)_6_ and 0.1 M KCl was observed at about 0.25 V, which gradually increased upon successive addition of DA. It implied that the electrospun nanofiber films could catalyze the oxidation of DA and lead to the electron transfer kinetics for the DA redox [38,53]. This phenomenon was probably due to the interaction of quinoid structures in PANI and PABA with phenyl structures of DA [13,38,54]. Table 2 shows the performances including sensitivity, limit of detection (LOD), limit of quantitation (LOQ) and linear range of the fabricated electrospun nanofiber films. The sensitivities of both PANI/f-CNTs and PABA/f-CNTs composite films are higher than their corresponding conducting polymers, whereas the LOD, LOQ and linear range of the electrospun composite films were lower than the polymer films. It could obviously be seen that the presence of f-CNTs in the nanofiber films can enhance the performances of conducting polymers. The performance improvement was due to the doping effect of the f-CNTs on the PANI and PABA structures, which could be confirmed from the XPS results. Moreover, the carboxylic acid groups on the surface of f-CNTs could enhance the electrocatalytic activity towards detection of DA on the PANI/f-CNTs and PABA/f-CNTs composites, which was possibly due to the hydrogen bonding interaction of carboxylic acid groups of f-CNTs with amine groups of DA [55]. As seen in Table 2, the sensitivity of the PABA/f-CNTs was higher than PANI/f-CNTs. It could be explained from the ratio of –N=/-NH- peak in XPS result, which, related to doping level of f-CNTs on PANI or PABA, was observed to be higher in PABA/f-CNTs than PANI/f-CNTs. The other reason is possibly from the amine group of PABA could further form covalent bond with the electro-oxidized form of DA known as dopaminechrome [38]. The proposed mechanism for detection of DA using PABA/f-CNTs electrospun film is illustrated in Scheme 2. The comparison of the fabricated electrospun nanofibers of PANI/f-CNTs and PABA/f-CNTs composites in this study with previous related studies is presented in Table 3. The PANI/f-CNTs and PABA/f-CNTs composites showed a lower linear range than other previous studies. This could imply that the developed DA biosensors utilize for detection of DA at low concentration.

### 3.3. Selectivity, Reproducibility, Repeatability and Stability Studies 

**Figure 7:**
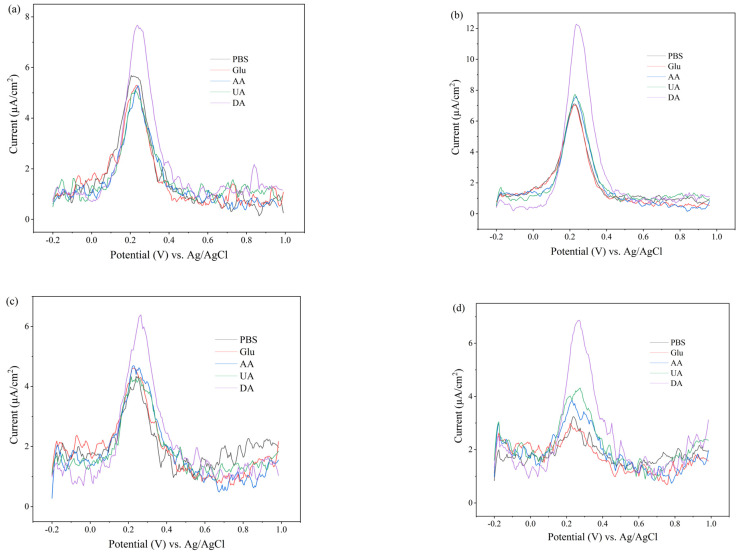
Differential pulse voltammograms of (**a**) PANI, (**b**) PANI/f-CNTs, (**c**) PABA and (**d**) PABA/f-CNTs electrospun nanofiber films upon addition 1 mM of common interferences (Glu, AA and UA) in PBS solution containing 0.5 mM K_3_Fe(CN)_6_ and 0.1 M KCl.

To study the selectivity of the electrospun nanofiber films, the common interferences, i.e., Glu, AA and UA in high concentrations (1 mM) were used for this study. Figure 7 shows the DPV responses of the electrospun nanofiber films upon addition of Glu, AA, UA and DA. It can be observed that the DPV responses upon injection of all common interferences were not significantly different from the initial DPV response of the electrospun nanofiber films in the PBS buffer solution containing 0.5 mM K_3_Fe(CN)_6_ and 0.1 M KCl. However, after injection of DA, the DPV responses of the electrospun nanofiber films obviously increased. These results indicated that the electrospun nanofiber films present good selectivity upon the addition of common interferences. In addition, other catecholamines such as serotonin, norepinephrine and epinephrine could also affect the detection of DA [38]. However, the low level of serotonin (5–23 ng/mL), norepinephrine (15–80 ng/mL) and epinephrine (0–20 ng/mL) in urine could not affect the detection of DA (52–480) in real samples [57,58]. Furthermore, in order to evaluate the reproducibility, the DPV responses of three similar independenly electrospun nanofiber films were measured upon the addition of 1 mM DA into PBS solution containing 0.5 mM K_3_Fe(CN)_6_ and 0.1 M KCl. All electrospun nanofiber electrodes exhibited consistent DPV current responses with low relative standard deviation (RSD) values of 0.687% for PANI, 0.243% for PANI/f-CNTs, 0.097% for PABA and 0.381% for PABA/f-CNTs which indicated the great reproducibility performance of all fabricated electrospun nanofiber films.

**Figure 8:**
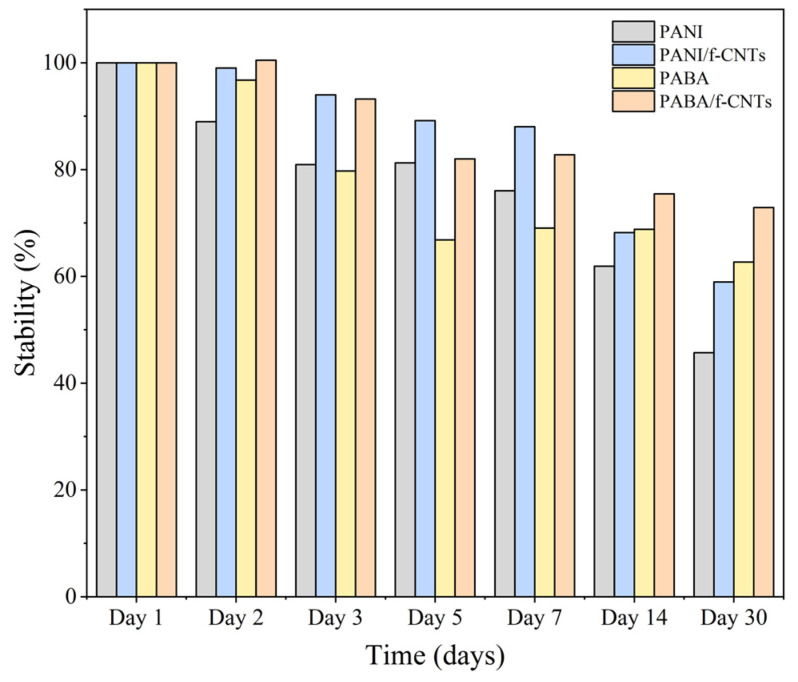
Stability histograms of the fabricated electrospun nanofibers of PANI/f-CNTs and PABA/f-CNTs composite films upon electrochemical detection of 1 mM DA.

The repeatability was assessed by successive addition of 1 mM DA into the electrospun nanofiber working electrodes in PBS solution containing 0.5 mM K_3_Fe(CN)_6_ and 0.1 M KCl for 10 cycles of DPV scanning. The PABA exhibited repeatability at 6 cycles, whereas PANI, PANI/f-CNTs and PABA/f-CNTs exhibited repeatability at 7 cycles (Figure S1). In addition, the stability was monitored by measuring the DPV peak currents by adding 1 mM DA in PBS solution containing 0.5 mM K_3_Fe(CN)_6_ and 0.1 M KCl for 1 cycle. The electrospun nanofiber films were stored at room temperature without humidity for up to 30 days. As seen in Figure 8, the PANI/f-CNTs and PABA/f-CNTs films presented the exact DPV responses, whereas the PABA film presented only a 3% decrease in DPV response after 2 days. The DPV response was obviously changed to below 80% after 3 days for the PABA film. The DPV responses dropped below 90% after 2 days for the PANI film and after 3 days for the PANI/f-CNTs and PABA/f-CNTs films. After 14 days, the DPV responses of all fabricated electrospun nanofiber films were lost below 80%. A decrease in DPV response indicated the degradation of the electrospun nanofibers upon storage. The stability improvement of the composite films could imply to the incorporation of the f-CNTs with the PANI and PABA. Thus, the fabricated electrospun nanofibers of PANI/f-CNTs and PABA/f-CNTs composites exhibited great performances with acceptable reproducibility, repeatability and stability for use as a label-free electrochemical dopamine biosensor.

### 3.4. Application of the Obtained Label-Free DA Biosensor in Artificial Urine

After obtaining the calibration curves as shown in the insets of Figure 6, the detection of DA in artificial urine was examined to verify the reliability of this study in practical application. The electrospun nanofibers of PANI/f-CNTs and PABA/f-CNTs composites were employed as working electrodes for label-free electrochemical detection of DA (0.15, 0.25, 0.35 µM). The electrochemical measurement was performed using the potential range of −0.2–1.0 V at a scan rate of 5 mV/s in 5% *v*/*v* of artificial urine in PBS solution containing 0.5 mM K_3_Fe(CN)_6_ and 0.1 M KCl using DPV technique. The percentage recovery was obtained from DPV peak currents of each concentration using three similar independent electrodes as shown in Table 4 [16,59]. The comparison with other published works [60,61,62,63,64] is presented in Table S1. As seen in the table, the developed electrodes in this work showed similar recovery values with lower RSD values. It could be seen that the developed label-free electrochemical dopamine biosensor based on the electrospun nanofibers of PANI/f-CNTs and PABA/f-CNTs composites demonstrated accurate results, which might be applicable for real medical sample analysis in the future.

## 4. Conclusions

The sensitive and selective label-free electrochemical DA biosensor based on the electrospun nanofibers of PANI and its aminated derivative, i.e., PABA composited with f-CNTs were successfully developed and fabricated for detection of DA. Prior to use as a DA biosensor, the electrochemical characteristics from the CV experiment and characterization results from ATR-FTIR spectroscopy, XPS, SEM and TEM techniques were obtained to confirm the existence of f-CNTs in the PANI and PABA composites. The presence of f-CNTs in the PANI and PABA utilized the greater electron transfer in the composites, which led to increasing of the performances, including sensitivity, selectivity, LOD and linear range of the obtained DA biosensors. From DPV responses, these electrochemical DA biosensors exhibited the sensitivity of 6.88 µA·cm^−2^·µM^−1^ for PANI/f-CNTs and 7.27 µA·cm^−2^·µM^−1^ for PABA/f-CNTS in the linear range of 50–500 nM (R^2^ = 0.98) with a limit of detection (LOD) of 0.0974 µM and 0.1554 µM, respectively. In addition, the developed electrochemical DA biosensors also showed the good reliability under detection of DA in artificial urine. Hence, the developed electrochemical DA biosensors based on the electrospun nanofibers of PANI/f-CNTs and PABA/f-CNTs composites demonstrated the capability for application in real medical sample analysis in the future.

## Figures and Tables

**Table 1:** Electrochemical surface area (cm^2^) with average difference between anodic and cathodic peak voltage (V) of the fabricated electrospun nanofiber films.

	PANI	PANI/f-CNT	PABA	PABA/f-CNT
A_anodic_	0.0104	0.0218	0.0096	0.0154
A_cathodic_	−0.0374	−0.0392	−0.0334	−0.0372
ΔE_a,c_	0.279	0.266	0.346	0.297

**Table 2:** Performances of the PANI/f-CNTs and PABA/f-CNTs composite films upon detection of dopamine.

	Sensitivity (µA·cm^−2^·µM^−1^)	LOD (µM)	LOQ (µM)	Linear Range (µM)
PANI	2.560	0.4542	1.5140	1.3–1.9
PANI/f-CNTs	6.876	0.0974	0.3245	0.05–0.5
PABA	2.809	0.4178	1.3926	1.1–1.5
PABA/f-CNTs	7.269	0.1554	0.5179	0.05–0.5

**Table 3:** Comparison of electrochemical dopamine biosensors based on conducting polymers/carbon nanomaterial composites.

Electrode	Linear Range (µM)	Sensitivity (µA·cm^−2^·µM^−1^)	LOD (µM)	References
PANI/CQDs	10–90	0.00802	0.1013	[13]
poly-β-CD(f-MWCNTs)/PANI	2–24	26.79	0.0164	[16]
GO/PEDOT:PSS	-	69.3	0.008	[20]
SPANI/CNSs	0.50–1780	1.139	0.0152	[25]
PANI-GO/GCE	2–18	-	0.5	[54]
CD/PNAANI/CNT modified CPE	2–200	-	sub-µM	[56]
PANI/f-CNT	0.05–0.5	6.876	0.0974	This work
PABA/f-CNT	0.05–0.5	7.269	0.1554	This work

Abbreviations: carbon paste electrode (CPE); β-cyclodextrin (CD); poly(N-acetylaniline) (PNAANI); carbon nanotube (CNT); graphene oxide (GO), poly(3,4-ethylenedioxythiophene) (PEDOT); polystyrene sulfonate (PSS); glassy carbon electrode (GCE); polyaniline (PANI); functionalized multi-walled carbon nanotubes (MWCNTs); carbon quantum dots (CQDs); poly(3-aminobenzylamine) (PABA).

**Table 4:** Determination of DA in artificial urine (n = 3).

Electrode	Added Concentration (µM)	Measured Concentration (µM)	Recovery (%)	RSD (%)
PANI/f-CNTs	0.15	152.88	101.92	0.634
	0.25	247.52	99.01	0.691
	0.35	346.51	99.00	0.806
PABA/f-CNTs	0.15	150.67	100.45	0.968
	0.25	248.01	99.20	1.829
	0.35	348.91	99.69	0.801

## Data Availability

The original contributions presented in the study are included in the article/Supplementary Materials, further inquiries can be directed to the corresponding author/s.

## Supplementary Materials

The following supporting information can be downloaded at: https://www.mdpi.com/article/10.3390/bios14070349/s1, Figure S1: Differential pulse voltammograms of (a) PANI, (b) PANI/f-CNTs, (c) PABA and (d) PABA/f-CNTs electrospun nanofiber films upon addition of 1 mM DA for up to 10 cycles in PBS solution containing 0.5 mM K_3_Fe(CN)_6_ and 0.1 M KCl. Table S1: Comparison of detection of dopamine in urine sample [60,61,62,63,64].
