# Supplementary material for: Label-Free Electrochemical Dopamine Biosensor Based on Electrospun Nanofibers of Polyaniline/Carbon Nanotube Composites
Source: Biosensors (Basel). 2024 Jul 18;14(7):349. doi: 10.3390/bios14070349 (PMC11275224; doi:10.3390/bios14070349)
Supplement: Supplementary file 1 [file biosensors-14-00349-s001.zip › biosensors-3059270-supplementary.pdf]

Label-Free Electrochemical Dopamine Biosensor Based on Electrospun Nanofibers of Polyaniline/Carbon Nanotube Composites

Chanaporn Kaewda and Saengrawee Sriwichai *

Department of Chemistry, Faculty of Science, Chiang Mai University

*E-mail: saengrawee.s@cmu.ac.th

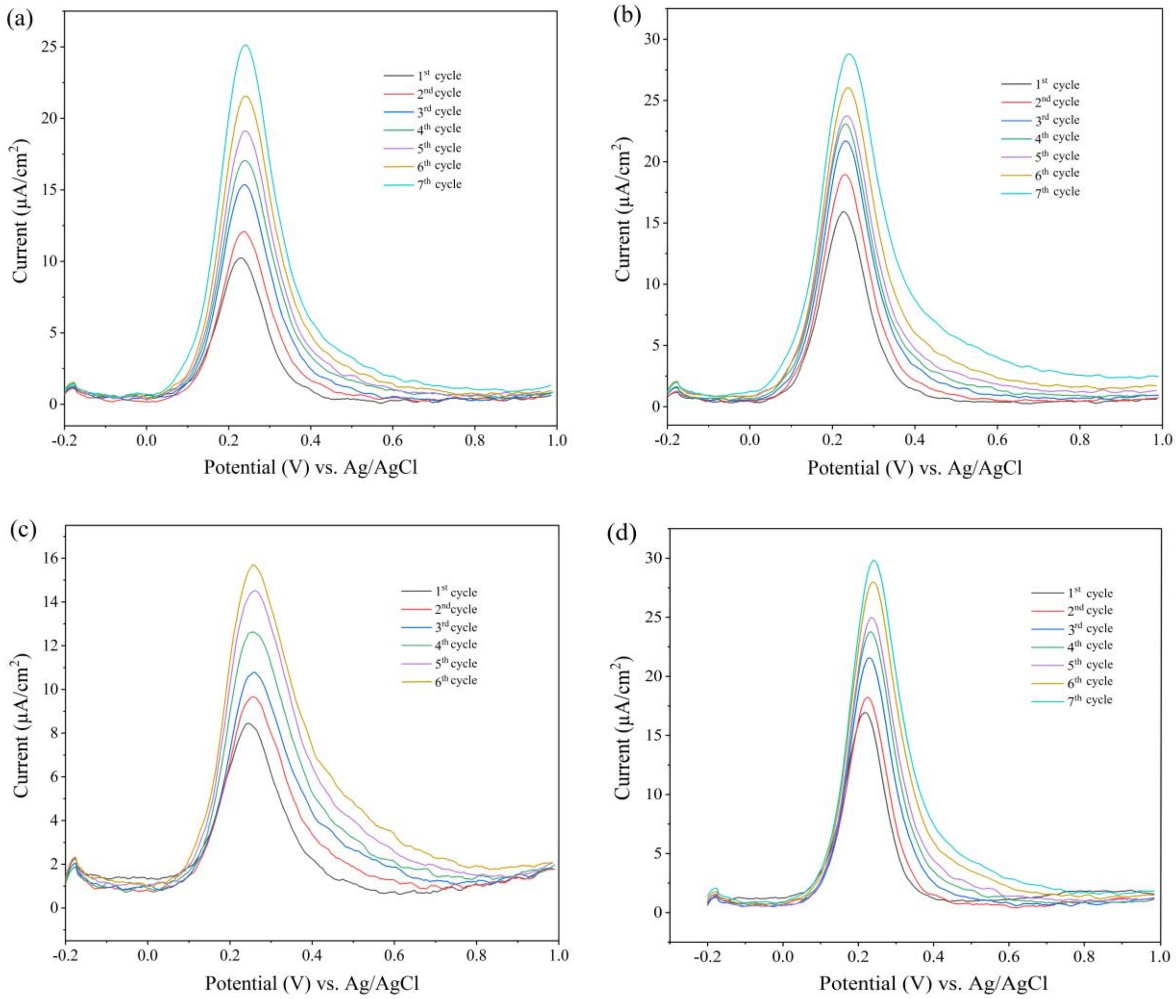

Figure S1. Differential pulse voltammograms of (a) PANI, (b) PANI/f-CNTs, (c) PABA and (d) PABA/f-CNTs electrospun nanofiber films upon addition of 1 mM DA for up to 10 cycles in PBS solution containing 0.5 mM $K_3Fe(CN)_6$ and 0.1 M KCl.

Table S1. Comparison of detection of dopamine in urine sample.

Electrode	Added (µM)	Found (µM)	%Recovery	%RSD	Ref.
Nano-Cu/PPy/GCE	10.50	10.37	98.8	1.92	[1]
	15.70	16.00	101.9	1.99	
	21.00	21.32	101.5	1.97	
MBIP	100	93±5	93	5.4	[2]
	200	208±8	104	3.8	
	500	496±11	99	2.2	
PL-LEU/DNA/GCE	10.0	10.2	102.0	1.6	[3]
	10.0	9.93	99.3	2.3	
	10.0	10.3	103.0	1.9	
PEDOT-Au_{nano}/Au in presence of SDS	1.00	1.01	101	1.64	[4]
	5.00	5.07	101.4	1.81	
	10.0	9.94	99.4	0.687	
	20.0	19.7	98.5	3.03	
	35.0	34.9	99.7	1.73	
PEDOT/Pt electrode in presence of SDS	4.00	3.99	99.8	-	[5]
	6.00	5.89	98.2	-	
	10.0	10.01	101.0	-	
	15.0	14.98	99.9	-	
PANI/f-CNTs	0.15	152.88	101.92	0.634	This work*
	0.25	247.52	99.01	0.691	
	0.35	346.51	99.00	0.806	
PABA/f-CNTs	0.15	150.67	100.45	0.968	
	0.25	248.01	99.20	1.829	
	0.35	348.91	99.69	0.801	

*Artificial urine was used in this work.

Abbreviations: polypyrrole (PPy), glassy carbon electrode (GCE), Multiple walled carbon nanotubes (MWCNTs), platinum (Pt), laccase (LAc), molecularly bioimprinted polymer (MBIP), poly(L-leucine) (PL-LEU), poly(3,4-ethylene-dioxythiophene)(PEDOT), gold nanoparticles (Au_{nano}), sodium dodecyl sulfate (SDS).

Reference

[1] Ulubay, S.; Dursun, Z. Cu Nanoparticles Incorporated Polypyrrole Modified GCE for Sensitive Simultaneous Determination of Dopamine and Uric acid. *Talanta* **2010,** *80,* 1461–1466.

[2] Rezaei, B.; Boroujeni, M. K.; Ensaf, A. A. Fabrication of DNA, *o*-Phenylenediamine, and Gold Nanoparticle Bioimprinted Polymer Electrochemical Sensor for the Determination of Dopamine. *Biosens. Bioelectron.* **2015,** *66,* 490–496.

[3] Zheng, X.; Guo, Y.; Zheng, J.; Zhou, X.; Li, Q.; Lin, R. Simultaneous Determination of Ascorbic Acid, Dopamine and Uric Acid using Poly(L-leucine)/DNA Composite Film Modified Electrode. *Sens. Actuator B-Chem.* **2015,** *1,* 1–30.

[4] Atta, N. F.; Galal, A.; El-Ads, E. H. Gold Nanoparticles-Coated Poly(3,4-Ethylene-Dioxythiophene) for the Selective Determination of Sub-Nano Concentrations of Dopamine in Presence of Sodium Dodecyl Sulfate. *Electrochim. Acta* **2012,** *69,* 102–111.

[5] Atta, N. F.; Galal, A.; Ahmed, R. A. Poly(3,4-Ethylene-Dioxythiophene) Electrode for the Selective Determination of Dopamine in Presence of Sodium Dodecyl Sulfate. *Bioelectrochemistry* **2011**, *80*, 132–141.
